# Usefulness of presepsin for the early detection of infectious complications after elective colorectal surgery, compared with C-reactive protein and procalcitonin

**DOI:** 10.1038/s41598-022-06613-w

**Published:** 2022-03-10

**Authors:** Erika Amanai, Kishiko Nakai, Junichi Saito, Eiji Hashiba, Takuya Miura, Hajime Morohashi, Yoshiyuki Sakamoto, Akio Mikami, Kenichi Hakamada, Kazuyoshi Hirota

**Affiliations:** 1 Department of Anesthesiology, Hirosaki University Hospital, 5 Zaifu-cho, Hirosaki, Aomori 036-8562 Japan; 2 Division of Intensive Care Unit, Hirosaki University Hospital, Hirosaki, Japan; 3 Department of Gastroenterological Surgery and Pediatric Surgery, Hirosaki University Graduate School of Medicine, Hirosaki, Japan; 4 Central Clinical Laboratory, Hirosaki University Hospital, Hirosaki, Japan; 5 Department of Anesthesiology, Hirosaki University Graduate School of Medicine, Hirosaki, Japan

**Keywords:** Biomarkers, Medical research

## Abstract

Infectious complications remain a major clinical problem in colorectal surgery. Presepsin has been reported to be a useful marker to diagnose sepsis, similar or superior to procalcitonin (PCT) and C-reactive protein (CRP). The aim of this study was to assess the diagnostic value of presepsin in the early detection of infectious complications after elective colorectal surgery, compared with CRP and PCT. This study was a prospective observational study. Patients of age > 18 who underwent elective colon resections were enrolled. Blood samples were collected just before surgery and on postoperative day (POD) 1, 2, 3, 4, and 6 to measure plasma levels of biomarkers. We evaluated the association between circulating biomarkers and infections. A total of 114 patients were examined, and 27 patients (23.7%) developed infectious complications. CRP and PCT markedly increased from POD 1 to POD 3 and then gradually decreased toward POD 6 in both groups, but the trends of the decrease in the infected group were blunt, compared with those in the non-infected group. On the other hand, presepsin did not show major changes just after surgery, but it increased on POD 4 and POD 6, when the complications occurred. Monitoring the presepsin trends after colorectal surgeries could be helpful to detect postoperative infectious complications.

**Trial registration:** UMIN000025313. Registered on 17 December 2016.

## Introduction

Infectious complications of surgery are associated with increased morbidity, length of hospitalization, and health care costs^1,2^. They are a particular problem in colorectal surgery, a procedure with disproportionately high rates of infectious complication^3,4^. Early detection and optimal treatment of infectious complications are crucial to improving mortality and morbidity^4,5^. C-reactive protein (CRP) is probably the most widely used inflammatory biomarker in hospitals worldwide. CRP has been studied as a useful predictor of infectious complications after colorectal resections. Platt et al. reported that CRP measurement on postoperative day (POD) 3 was clinically useful in predicting infectious complications, but its sensitivity and specificity are not very high^6^. Procalcitonin (PCT), a 116-amino acid peptide, is an inflammatory biomarker with a shorter half-life than CRP. Unfortunately, PCT levels are elevated in numerous noninfectious conditions such as trauma, burns and surgical procedures themselves, making accurate interpretation of PCT elevation difficult^7^.

Presepsin is a soluble N-terminal fragment of the cluster of the differentiation marker protein CD14 (sCD14-ST), which is released into the circulation during monocyte activation upon the recognition of lipopolysaccharide from infectious agents^8^. Several studies have confirmed the usefulness of presepsin as a marker for the diagnosis of sepsis^9,10^. In studies, plasma presepsin concentrations have been linked the severity of sepsis^8,11^ and its outcome^12^. Moreover, it has been reported that presepsin levels are not susceptible to severe trauma, burn or invasive surgical procedures, and that, as an early marker of mortality for critically ill patients, it showed better prognostic performance than other biomarkers^13,14^. However, it remains unknown whether presepsin is useful for the early detection of infectious complications after elective colorectal surgery.

The aim of this study was to assess the diagnostic value of presepsin levels in the early detection of infectious complications after elective colorectal surgery. We compared presepsin in this role with CRP and PCT.

## Methods

### Study design

This study was performed as a prospective, single-center, observational study. All study participants provided informed consent. The study protocol was approved by the Ethics Committee of Hirosaki University Graduate School of Medicine and registered in a publicly accessible database, the UMIN clinical Trial Registry (UMIN000025313). All methods were performed in accordance with relevant guidelines and regulations.

Patients were enrolled from January 9, to December 31, 2017. Patients 18 years or older who had undergone elective colorectal surgery were enrolled. Patients were excluded if they had obvious signs of infection before surgery or end-stage kidney disease (defined by the Risk-Injury-Failure-Loss-Endstage renal disease classification criteria), as the latter is known to affect presepsin concentrations^15–17^. Perioperative managements based on the enhanced recovery after surgery (ERAS) protocols have been performed during the study period as follows. Patients could have clear liquids up to 2 h before the induction of anesthesia. Anesthesia was induced and maintained with propofol, remifentanil, ketamine, and rocuronium. After the induction of anesthesia, ultrasound-guided single-shot abdominal trunk blocks were performed with 40–60 ml of 0.2–0.25% levobupivacaine. The antibiotic prophylaxis consisted of cefotiam hydrochloride 1 g within 60 min prior to incision and every 3 h after surgery for 24 h.

### Data collection and definition of complications

Clinical data were obtained during an initial patient interview and subsequent review of medical documentation. Patient demographic and perioperative data were entered into an electronic databank.

Postoperative infectious complications were classified as surgical site infections (SSIs) and remote infections. SSIs included wound infection, intra-abdominal infection, and anastomotic leakage. Remote infections such as pneumonia, urinary tract infections and central line infections were often exogenous and occurred at sites not directly associated with the surgical procedure^18,19^. Infectious complications were diagnosed by surgeons. Patients underwent postoperative diagnostic tests or treatments only in case of symptoms or signs of infectious complications. Anastomotic leakage was defined as leakage at the site of surgical anastomosis verified either radiologically or upon relaparotomy. The severity of anastomotic leakage was determined using the criteria established by the International Study Group of Rectal Cancer (ISREC) and classified as grades A, B, and C^20^. Intra-abdominal infection was verified by surgical drainage, imaging study, or by relaparotomy. Wound infection was defined as the presence of pus, either discharged spontaneously or requiring drainage. Pneumonia was diagnosed by the presence of new pulmonary infiltration on chest radiography, accompanied by clinical symptoms and signs. Urinary tract infection was diagnosed by clinical symptoms with a positive urine sediment analysis. Central line infection was diagnosed by clinical symptoms with positive blood cultures and cultures from the catheter tip^21,22^.

### Circulating biomarker measurement

Blood samples were collected through an arterial line just before surgery and on POD 1, 2, 3, 4, and 6 to measure plasma levels of presepsin, CRP, and PCT.

Presepsin concentrations were determined by a chemiluminescent enzyme immunoassay. The lower limit of detection was 20 pg/mL. PCT concentrations were determined by an electrochemiluminescent immunoassay, according to the manufacturer’s instructions. The lower limit of detection was 0.02 ng/mL. CRP concentrations were measured by a latex coagulation detection method with a nephelometer. The lower limit of detection was 0.02 mg/dL.

### Outcomes and statistical analysis

We evaluated the association between circulating biomarkers and infections. More specifically, we assessed the diagnostic value of postoperative presepsin levels in the early detection of infectious complications after elective colorectal surgery, compared with PCT and CRP levels.

In designing the study, we calculated the necessary sample size of at least 95 patients to achieve 95% statistical power at a type I error probability of 0.05% to detect an AUC of 0.8, and a rate of SSI or 13%^2,23–25^. Anticipating a 20% loss to follow-up, this number was increased to 114 patients. All statistical calculations were performed using GraphPad Prism version 6.0 (GraphPad Software, La Jolla, CA) and EZR (Saitama Medical Center, Jichi Medical University, Saitama, Japan), which is a graphical user interface for R (The R Foundation for Statistical Computing, Vienna, Austria). Student’s t-test was used for the comparison of two groups of parameters that were normally distributed. The Mann–Whitney U test was used to compare differences between two independent groups not normally distributed. Changes of biomarker concentrations over time in both groups were compared using two-way analysis of variance (ANOVA) with repeated measures followed by Bonferroni’s multiple comparison test on log-transformed data when appropriate. Predictive performance was assessed by the area under the time-dependent receiver operating characteristic (ROC) curves; the cutoff values, sensitivities, specificities were calculated. The relations among the calculated cutoff values of a circulating biomarker, clinically relevant variables, and the presence or absence of infectious complications was assessed with Cox proportional hazards models. Clinically relevant variables that were recognized as risk factors for infectious complications after colorectal surgery, i.e., those with an ‘American Society of Anesthesiologists (ASA) physical status classification ≥ 3’ and ‘not laparoscopic surgery’ were included in the model^26^. We obtained hazard ratios and 95% confidence intervals for each variable. A p value < 0.05 was accepted as significant.

### Ethics approval and consent to participate

This study protocol was approved by our university ethical committee and registered in a publicly assessable database, the UMIN clinical Trial Registry (in the Japan Primary Registries Network; UMIN000025313).

## Results

### The demographic data of the patients (Table 1)

**Table 1:** Clinical characteristics of patients with and without infectious complications.

	Non-infected	Infected	P Value
	N = 87	N = 27	
Age, yr	69.1 ± 11.5	63.7 ± 13.2	**0.044**
Male, %	62 (71)	17 (62)	0.414
BMI (kg/m^2^)	23.2 ± 3.7	22.7 ± 2.7	0.592
ASA PS I/II/III	0/65/22	2/20/5	**0.033**
**Diagnosis**			
Cancer, N (%)	82 (94.2)	26 (96.3)	0.677
**Location of the disease**			
Rectum, N (%)	43 (49.4)	20 (74.0)	**0.024**
**Surgical intervention**			0.107
Right hemicolectomy, N (%)	24 (27.5)	3 (11.1)	
Sigmoidectomy, N (%)	16 (18.3)	4 (14.8)	
Total colectomy, N (%)	3 (3.4)	0 (0.0)	
Anterior resection, N (%)	23 (26.4)	6 (22.2)	
APR, N (%)	14 (16.0)	8 (29.6)	
ISR, N (%)	6 (6.8)	6 (22.2)	
**Surgical approach**			
Laparoscopy, N (%)	61 (70.1)	11 (40.7)	**0.006**
Length of hospital stay (d)	12.6 ± 7.1	24.5 ± 29.6	** < 0.001**

Data: mean ± SD, *BMI* body mass index, *ASA PS* American Society of Anesthesiologists Physical Status, *ISR* intersphincteric resection, *APR* abdominoperineal resection.

Significance values are in Bold.

A total of 114 patients were included in this study, and 27 patients (23.7%) developed infectious complications. The clinical characteristics of the 114 patients are shown in Table 1. The complications included 11 anastomotic leakages, 13 intra-abdominal infections and three wound infections. Among those 27 patients with complications, one patient coincidentally had a urinary tract infection beside intra-abdominal infection. According to the severity grading of the ISREC, there were five grade B patients and six grade C patients. Six patients required additional operations: four ileo-colostomies, one Hartmann resection and one re-suturing of the anal region. The median interval between surgery and the diagnosis of infection was 5 days (Min–Max days: 3–9 days).

The patients who underwent rectal resections had significantly more complications than the patients who did not undergo rectal surgery (74.0% vs 49.4%, p = 0.024). The length of hospital stay was significantly longer in patients with complications than in patients without complications (24.5 ± 29.6 vs. 12.6 ± 7.1, p < 0.001), implying higher costs of medical treatment for patients with complications. There was no readmission due to morbidities within the first 30 days after surgery.

### Time course of presepsin, CRP and procalcitonin levels during the study period

**Figure 1:**
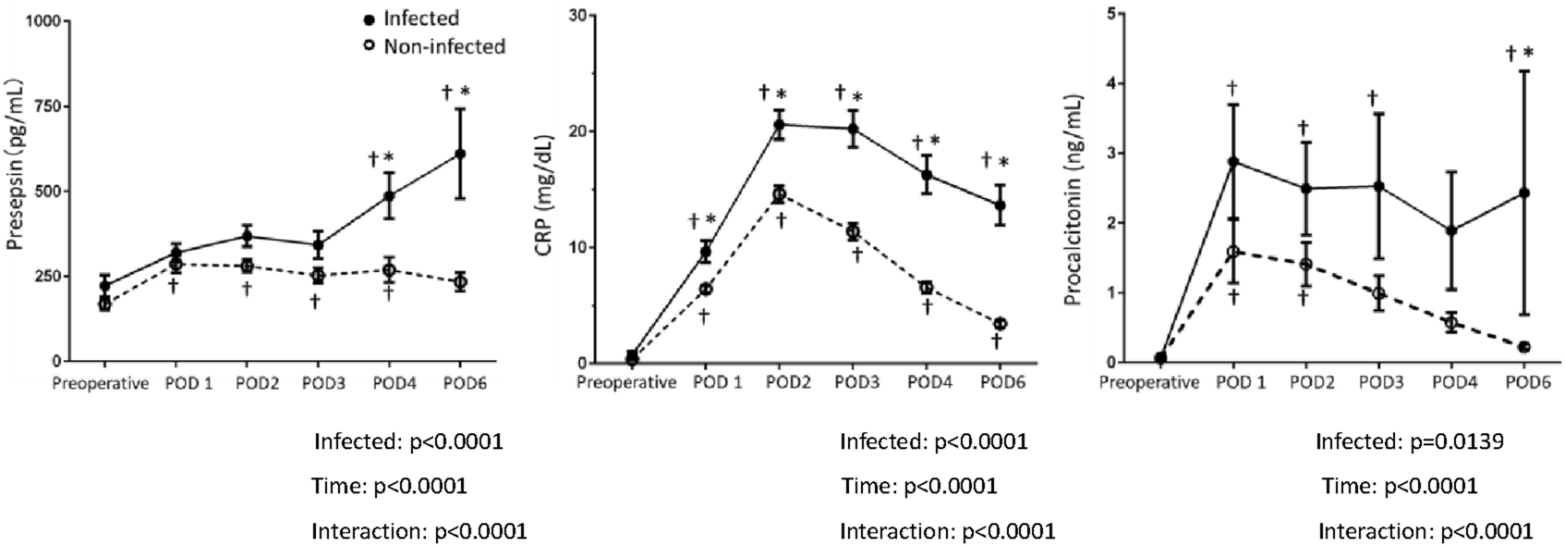
Time course of plasma concentrations of presepsin, CRP and procalcitonin. Plasma concentrations of presepsin, CRP and procalcitonin before surgery and on postoperative day 1, 2, 3, 4, and 6. Data are shown as mean and SEM. Two-way analysis of variance (ANOVA) with repeated measures was done on log-transformed data. There were significant differences between groups for presepsin, CRP, and procalcitonin concentrations. *p < 0.05 between groups. ^†^p < 0.05 versus preoperative.

Changes in the three biomarkers are shown in Fig. 1. There were significant differences in presepsin, CRP and PCT concentrations between the infected and non-infected groups (each P value for time-infected interaction was less than 0.0001). CRP and PCT significantly and markedly increased from POD 1 to POD 3 and then gradually decreased toward POD 6, but the decreases in the infected group were more gradual than those in the non-infected group.

On the other hand, presepsin did not increase in either group within the first 3 days after the surgery, but the infected group showed a significant increase on POD 4 and POD 6, compared with the non-infected group. The shapes of the trend of presepsin were clearly different from those of the other two markers, CRP and PCT.

### Prognostic accuracy of presepsin, CRP and PCT

The results of time-dependent ROC analysis defining the area under the curve (AUC) of the three biomarkers for infectious complications are shown in Table 2. Time-dependent ROC analysis to predict infectious complications revealed that the best accuracy was obtained on POD 6 for all biomarkers. The cut-off values, sensitivities, specificities and AUCs are also shown in Table 2. The AUC values of CRP, presepsin and PCT on POD 6 were 0.88, 0.79 and 0.74, respectively.

**Table 2:** time-dependent ROC curve predictions of presepsin, CRP and procalcitonin (PCT) values based on the presence or absence of infectious complications.

		Preoperative	POD1	POD2	POD3	POD4	**POD6**
Presepsin	Cut-off (pg/ml)	221	274	307	182	385	**294**
	AUC	0.65	0.64	0.70	0.67	0.73	**0.79**
	Sensitivity (%)	44.1	68.9	71.0	87.9	54.9	**70.0**
	Specificity (%)	83.2	64.7	73.2	43.1	87.8	**81.8**
CRP	Cut-off (mg/dl)	0.70	8.40	17.06	13.18	6.81	**7.30**
	AUC	0.54	0.71	0.73	0.80	0.86	**0.88**
	Sensitivity (%)	29.1	65.6	73.2	80.0	94.4	**79.6**
	Specificity (%)	89.7	78.2	66.9	72.4	64.6	**88.8**
PCT	Cut-off (ng/ml)	0.10	0.22	0.28	0.20	0.14	**0.08**
	AUC	0.47	0.61	0.62	0.63	0.68	**0.74**
	Sensitivity (%)	18.2	85.7	74.7	78.0	81.2	**91.2**
	Specificity (%)	92.0	39.8	52.3	53.4	54.4	**43.7**

Significance values are in Bold.

The relationships among the calculated cutoff values of a circulating biomarker, clinically relevant variables (‘ASA ≥ 3’ and ‘not laparoscopic surgery’) and the presence or absence of infectious complications was assessed with Cox proportional hazards models (Table 3). The cutoff values of CRP and presepsin on POD 1, 2, 3, 4, and 6 were independently related to the presence or absence of infectious complications.

**Table 3:** Multivariate COX models for the presence or absence of infectious complications.

		Cutoff value of biomarker	HR	95% CI	P Value
Presepsin	Preoperative	221	1.97	0.90–4.31	0.08
	POD 1	274	2.39	1.00–5.69	0.04
	POD 2	307	3.57	1.50–8.47	0.003
	POD 3	182	3.92	1.16–13.26	0.027
	POD 4	385	4.34	1.92–9.81	< 0.001
	POD 6	294	5.66	2.38–13.45	< 0.001
CRP	Preoperative	0.70	1.91	0.81–4.40	0.134
	POD 1	8.40	3.79	1.59–9.00	0.002
	POD 2	17.06	3.20	1.29–7.90	0.011
	POD 3	13.18	6.56	2.43–17.71	< 0.001
	POD 4	6.81	30.46	4.09–226.50	< 0.001
	POD 6	7.30	14.92	5.45–40.81	< 0.001
PCT	Preoperative	0.10	2.04	0.77–5.43	0.150
	POD 1	0.22	1.91	0.57–6.31	0.287
	POD 2	0.28	1.75	0.66–4.59	0.255
	POD 3	0.20	1.91	0.74–4.92	0.177
	POD 4	0.14	2.56	0.89–7.35	0.079
	POD 6	0.08	7.17	0.95–54.04	0.055

Risk is presented as hazard ratio and 95%conficence interval. ‘American Society of Anesthesiologist (ASA) physical status classification≧3’ and ‘not endoscope’ which were deemed important clinical variables were considered in the multivariable models.

## Discussion

This study showed the unique behaviors of presepsin in colorectal surgery patients with and without infectious complications, compared with CRP and PCT. CRP and PCT significantly increased immediately after the surgery and gradually decreased several days after the surgery regardless the infectious complications. On the other hand, presepsin did not show major changes just after surgery, but it increased on POD 4 and POD 6, when the infectious complications occurred.

CRP is an inflammatory biomarker that increases in response not only to infection but also to inflammation caused by non-infectious stimuli. It is widely used as a predictor of infectious complications after surgery. Platt et al.^6^ reported that CRP measurement on POD 3 is clinically useful for predicting infectious complications. PCT has been also reported to be useful as a septic biomarker^27^, but PCT levels are also known to be elevated in non-infectious conditions such as trauma, burn and even surgical procedures^7,28^. In this study the levels of mean PCT concentrations in both infected and non-infected groups were higher than 1 ng/ml, which was more than the reported cutoff values of 0.5 ng/ml for suspecting bacterial infections^28,29^. Overall, PCT may not be very useful for detecting infectious complications in colorectal surgery because PCT is so easily increased, the levels of increase vary from patient to patient, and the cutoff values of PCT during the study period were not significantly related to the presence or absence of infectious complications in the Cox proportional hazards models. In contrast to CRP and PCT, presepsin markedly increased only in response to the infectious insults after surgery, with good predictability shown by an AUC of 0.79 on POD 6. Presepsin has been shown to be released into the blood circulation during monocyte activation upon the recognition of lipopolysaccharides from infectious agents^8^. Several studies have confirmed the usefulness of presepsin as a biomarker for the diagnosis of sepsis^9,10^. Our study showed the usefulness of presepsin to detect infectious complications in colorectal surgery in line with those previously reported studies.

The cutoff value of presepsin to detect infection on POD 6 was 294 pg/ml, less than previously reported values. Popv and colleague^30^ reported that the cutoff value of presepsin to identify infection after cardiac surgery was 702 pg/ml, with a sensitivity of 72% and a specificity of 66%. Endo and colleagues^10^ reported that the cutoff value of presepsin to discriminate bacterial from nonbacterial infectious disease was 600 ng/l, with a sensitivity of 87.8% and specificity of 81.4%. On the other hand, Liu and colleagues^14^ reported that the cutoff value of presepsin for diagnosing sepsis was 317 pg/ml, with a sensitivity of 70.8% and specificity of 85.8%, and that the median [25th to 75th percentiles] presepsin concentration in healthy individuals was 130 [104, 179] pg/ml. Tsuchida and colleagues^31^ reported the cutoff values of presepsin for detecting bacterial infection and bacteremia in non-severe outpatients were 226 pg/ml (sensitivity 67%, specificity 66%) and 256 pg/ml (sensitivity 84%, specificity 70%), respectively. The latter two studies, as well as our own, suggest that the optimal cutoff value of presepsin to detect an infectious complication may not be as large as expected.

In our study, CRP had the highest sensitivity of 79.6% and specificity of 88.8% on POD 6, with an AUC of 0.88, which was higher than that of presepsin, suggesting that CRP might be better at detecting postoperative infections in colorectal surgery. However, the cutoff value of CRP was 7.30 mg/dl, which was not extraordinary high and much lower than the peak CRP level after surgery even in the non-infected group. In order to use the cutoff value of CRP in POD 6, it’s important to know its entire post-surgical trajectory.

In our hospital, patients whose post-operative course was uneventful were discharged 6 days after colorectal surgery, which might be a few days later than the other countries. Although length of hospital stay is dependent on the health care system in each country, it is universally important to diagnose infectious complications as early as possible in order to avoid inappropriate patient discharges and to minimize the morbidity due to the infections. The median interval between surgery and the diagnosis of infection was 5 days in this study. The biggest AUCs of the biomarkers were obtained on POD6, but the cutoff values of CRP and presepsin on POD 1, 2, 3 and 4 were independently related to the presence or absence of infectious complications in a multivariate analysis; that is, the CRP and presepsin cutoff values were independent explanatory factors of infectious complications from POD 1. The sensitivity and specificity of CRP on POD 4 were 94.4% and 64.6%, and the sensitivity and specificity of presepsin on POD 4 were 54.9% and 87.8%, respectively. Additionally, presepsin had a unique behavior that it did not show major changes just after surgery, but increased when the infectious complications were occurred. These results suggest that the measurements of CRP and presepsin could be useful to detect or deny infectious complications at an earlier point after colorectal surgery.

Our study has several limitations, including its single-center design. First, we did not examine the correlation between postoperative renal function and biomarker concentrations. We simply excluded patients with diagnosed end-stage kidney disease, knowing that renal function can affect presepsin concentrations. Even though there were no patients in this study who required renal replacement therapy during our observation, it was known that presepsin and PCT concentrations are significantly correlated with the serum creatinine concentration after cardiac surgery^32^. Thus, the postoperative course of renal function might have affected biomarker concentrations in this study. Second, blood samples were not collected on POD 5 when it was the median time period for diagnosis of infection in this study. It might be better to obtain blood samples on POD 5 to observe the time-dependent course. However, we expected the effectiveness of biomarker measurement in the early phase and took into consideration previous reports^6,23^.

## Conclusion

The trend of change in presepsin levels following colorectal surgery was distinct from those of CRP and PCT. Our data suggest that monitoring presepsin concentrations beside CRP after colorectal surgery is helpful for detecting early postoperative infectious complications.

## Data Availability

All data generated or analysed during this study are included in this published article.
